# A method for calculating the downslope sliding force of gently dipping bedding rock slopes while accounting for the uncertainty of rear-edge vertical fissures

**DOI:** 10.1371/journal.pone.0342903

**Published:** 2026-02-20

**Authors:** Zhenghang Ren, Junhao Liu, Rui Yang, Licheng Wu, Peng Zan, Haifu Tang

**Affiliations:** School of Civil Engineering, Guizhou University, Guiyang, China; Northeastern University, CHINA

## Abstract

The position of rear-edge vertical fissures in gently dipping bedding rock slopes is a critical factor in controlling their stability. However, the exact location of these fissures is often uncertain, which presents challenges for stability assessment and hazard prediction. Therefore, it is essential to develop a theoretical method capable of identifying the most unfavorable fissure position. Based on the geometric relationships and static equilibrium conditions of the unstable slope mass, this study systematically analyzes the water pressure distribution characteristics of rear-edge fissures under various water filling and outflow conditions. Four typical mechanical calculation models are constructed: (a) fissure filled with water and blocked outflow fissures, (b) fissure filled with water and unblocked outflow fissures, (c) considering only hydrostatic pressure, and (d) no water pressure scenario. By deriving the sliding force calculation formula and introducing an extremum criterion, the most critical fissure position and its corresponding maximum residual sliding force are determined. The results indicate that: (1) the width of the potential slip surface increases with the slope crest inclination (*α*) and slope height (*H*), but decreases as the bedding dip angle (*θ*) increases, (2) the geometric parameters (*H*, *α*, *θ*) have a significantly greater impact on the slip surface width than the shear strength parameters (*c*, *φ*) of the rock mass and (3) water pressure plays a significant role in altering the most unfavorable fissure position and serves as a critical hazard-inducing factor. Compared to traditional methods, which fix the fissure position at 1.5 times the slope height behind the slope face, the proposed method accurately identifies the most critical fissure location, effectively minimizing calculation errors. This study provides a more reliable mechanical model and computational foundation for stability analysis of gently inclined bedding rock slopes, offering direct guidance for disaster prevention design, risk management, and reinforcement strategies in similar slope engineering projects.

## Introduction

Gently inclined bedding rock slopes are widely distributed across central and western regions, exhibiting complex formation mechanisms and posing significant challenges for identification [1]. Under natural conditions, the slip plane inclination of such slopes typically falls below 45°, exhibiting high stability and a low frequency of large-scale landslide events [2–4]. Consequently, they are often overlooked in geological hazard surveys and early warning systems. However, recent increases in engineering activities have destabilized such slopes, causing a marked rise in landslide events of gently inclined bedding rock slopes. Failures of this type are often characterized by their large scale and high destructive potential, readily resulting in significant casualties and substantial property loss [5–7]. Currently, statistical data from a large number of instability cases in bedded rock slopes show that the dip angles of sliding surfaces mainly lie between 10° and 40° [8,9]. The majority of these cases are concentrated between 15°and 25°, characteristic of typical low-angle sliding failures. In addition, several failure cases with even gentler angles, ranging from 8°to 10°, have been identified in the Eastern Sichuan area [10,11].

Under natural conditions, the bedding dip of gently inclined slopes is typically lower than the friction angle within the bedding plane, and rear-edge vertical fissures at the rear edge are commonly observed. These fissures generally form in the lower or trailing portions of the slope as a result of external disturbances such as excavation or rainfall infiltration. In engineering practice, uncertainties in the location and geometry of fissures, along with factors such as fissure water ingress and fissure propagation, exert a significant influence on slope stability [12–15]. Accurately identifying the position of rear-edge vertical fissures in bedding rock slopes remains a major challenge. Although techniques such as geological mapping, geophysical prospecting, and remote sensing monitoring [16–18] can be applied, these approaches are often costly and technically demanding. To overcome these difficulties, several theoretical computational methods have been proposed by researchers. Studies indicate that a simplified calculation model can determine the location of the rear crack corresponding to the maximum residual sliding force [19]. However, this method overlooks the effect of water filling in the rear crack. The influence of water filling in the rear-edge vertical fissure on the stability of bedded rock slopes is primarily manifested in the following two aspects [20–23]: (1) water reduces the friction strength of the sliding surface through physicochemical interactions, thereby decreasing the slope’s resistance to sliding; (2) groundwater seepage through rear-edge fissures and potential slip surfaces forms seepage pathways. The resulting mechanical effects primarily include the hydrostatic pressure on fissure surfaces and the uplift pressure on slip surfaces. Regarding the hydraulic distribution hypothesis in rock slopes (under the condition of unblocked discharge fissures), Hoek and Bray [24] proposed that the maximum water pressure occurs at the base of the rear-edge vertical fissure, noting that the safety factor against sliding under saturated conditions is approximately 70% lower than that in dry slopes. Research indicates that under groundwater forces, hydrostatic pressure in tension cracks and uplift pressure on slip surfaces significantly reduce the slope safety factor. In contrast, the effect of hydrodynamic pressure is slight [25,26]. Moreover, investigation into the hydraulic failure mechanism of bedding slopes has yielded a relationship connecting critical rainfall intensity with fissure water pressure [27,28]. Further research indicates that several factors greatly affect slope stability with water-filled cracks. These include the slip surface angle, slope height, crest angle, and water depth. In short, these studies confirm that fissure water pressure is closely linked to slope instability [29]. Therefore, existing studies often neglect the influence of water pressure or discuss only a single hydraulic condition when determining the location of trailing vertical fissures, which presents significant limitations in practical engineering applications. To ensure analytical results align closely with engineering reality, it is essential to systematically consider the effects of different hydraulic conditions on both the location and stability of these fissures.

Unlike previous studies, this work specifically focuses on the positional uncertainty of rear-edge vertical fissures. Because water-filling and drainage conditions (blocked or unblocked) strongly affect hydro-mechanical behavior, four representative scenarios are defined: (I) water-filled fissure with blocked drainage, (II) water-filled fissure with unblocked drainage, (III) water-filled fissure without drainage effects, and (IV) dry fissure without water pressure. An extremum-based criterion was employed to identify the maximum residual sliding force and the corresponding critical fissure position. The effects of key parameters—including crest inclination, bedding dip, internal friction angle, cohesion, and slope height—on the critical fissure location were systematically investigated. Validation through real engineering cases confirmed the accuracy and applicability of the proposed formulation. The findings provide a solid theoretical foundation for hazard prevention, risk management, and reinforcement design of gently inclined layered rock slopes.

## Mechanical modeling and fundamental assumptions

**Fig 1:**
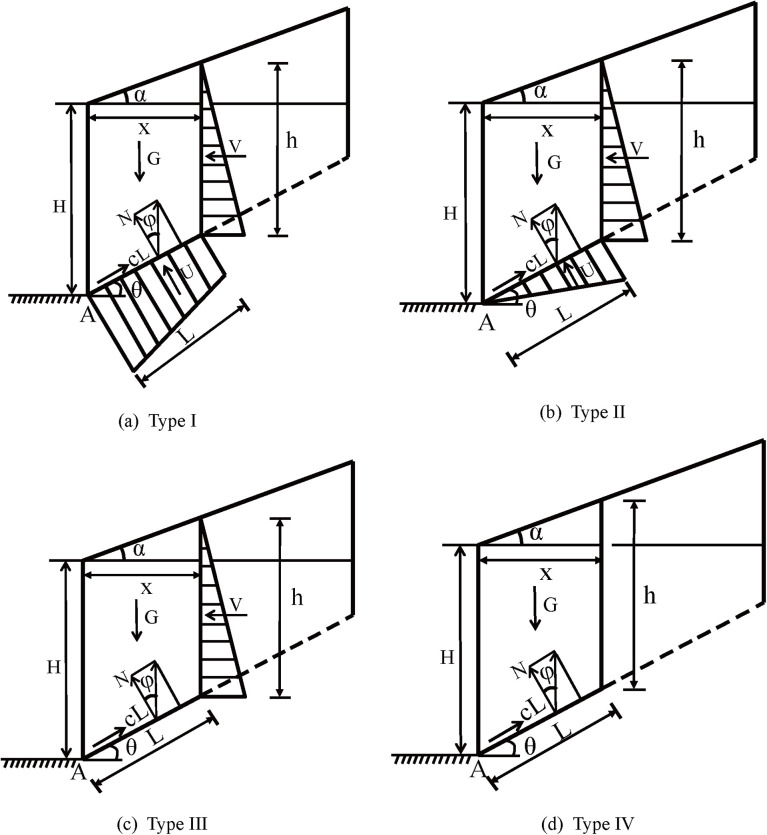
Residual sliding force models for four types of bedding rock slopes sliding along gently inclined outward-dipping structural planes.

Four residual sliding-force models were established for bedding rock slopes sliding along gently inclined, outward-dipping structural planes:(I) water-filled rear-edge vertical fissure with blocked drainage,(II) water-filled rear-edge vertical fissure with unblocked drainage,(III) water filling considered only, and(IV) dry condition without water pressure (Fig 1). Let L denote the length of the potential slip surface, H the slope height, and x the width of the potential sliding surface. The acting forces comprise the block self-weight (*G*), hydrostatic pressure (*V*) along the steeply dipping rear-edge vertical fissure, uplift pressure (*U*) along the gently dipping slip surface, normal reaction (*N*), frictional resistance (*N* tan *φ*), and cohesive resistance (*cL*) along the slip surface. Here, *θ* is the bedding dip of the gently inclined strata, *α* is the crest inclination angle, *c* the cohesive strength of the weak structural interface, *φ* its internal friction angle, and *h* the water-filled height within the rear-edge vertical fissure. The model is established under the following assumptions:

(1) The sliding mass is assumed to consist of homogeneous geomaterials.
(2) rear-edge vertical fissures are generally formed by external disturbances such as rainfall or excavation and are characterized by steep dips (>30^°^); hence, they are idealized as steeply dipping fissures in the model.
(3) The water level within the fissure is assumed to reach the intersection with the slip surface, indicating that the water-filled height is equal to the fissure depth extending to the slip plane.

## Analytical determination of the sliding force

### Formulation of the computational method

#### Type I—water-filled rear-edge vertical fissure with blocked drainage.

By resolving all acting forces along the slope inclination, the residual sliding force (Fr) can be expressed as follows:


$$F_{r}=F_{t}T-R$$ (1)

Based on the geometric relationships shown in Fig 1(a) and the static equilibrium conditions, the following equation is obtained:


T=Gsinθ+Vcosθ (2)

$$G=\frac{\text{1}}{\text{2}}\gamma (2H+(\tan\alpha -\tan\theta )x)x$$ (3)

$$cL=\frac{cx}{\cos\theta }$$ (4)

R=cL+(Gcosθ−Vsinθ−U)tanφ (5)

$$V=\frac{\gamma_{w}{\left(H-x\tan\theta +x\tan\alpha \right)}^{2}}{\text{2}}$$ (6)

$$U=\frac{\text{1}}{\text{2}}\gamma_{w}(2H-x\tan\theta +2x\tan\alpha )\frac{x}{\cos\theta }$$ (7)

In this equation, Ft denotes the safety factor defined for slope classification (for Grade I slopes, Ft = 1.35). Adopting this value as a general assumption allows for the evaluation of slope stability under the most unfavorable conditions. Grade I slopes typically require high stability standards to ensure that their safety factors meet or exceed the specified safety thresholds, thereby preventing potential instability. T is the sliding force; R is the anti-sliding force; V represents the hydrostatic pressure acting within the steeply dipping rear-edge vertical fissure; and U denotes the uplift pressure acting along the gently inclined slip surface. γ represents the unit weight of the rock mass, which is the weight per unit volume of the rock and typically ranges from 22 to 28 kN/m³. Similarly, γ_w_ represents the unit weight of water, defined as the weight per unit volume of water, typically taken as 10 kN/m³.By substituting Eqs. (2)–(7) into Eq. (1), the residual sliding force Fr_1_ is obtained as follows:


$$Fr_{\text{1}}=K_{1}x^{2}+K_{2}x+\frac{1.35}{\text{2}}\gamma_{w}H^{2}\cos\theta \;+\;\frac{\text{1}}{\text{2}}\gamma_{w}H^{2}\sin\theta \tan\varphi$$ (8)

$$\begin{array}{c} K_{1}=\frac{1.35}{\text{2}}\gamma_{w}(\tan\alpha -\tan\theta {)}^{2}\cos\theta +\frac{\text{1}}{\text{2}}\gamma (\tan\alpha -\tan\theta )\cos\theta (1.35\tan\theta -\tan\varphi )\\ \;+\;\frac{\text{1}}{\text{2}}\gamma_{w}(\tan\alpha -\tan\theta {)}^{2}\sin\theta \tan\varphi +\frac{\text{1}}{\text{2}}\gamma_{w}\frac{(2\tan\alpha -\tan\theta )\tan\varphi }{\cos\theta } \end{array}$$ (9)

$$\begin{array}{c} K_{2}=1.35H\gamma \sin\theta \;+\;1.35\gamma_{w}H(\tan\alpha -\tan\theta )\cos\theta -\frac{c}{\cos\theta }-H\gamma \tan\varphi \cos\theta \\ \;+\;\frac{\gamma_{w}H\tan\varphi }{\cos\theta }+\gamma_{w}H\sin\theta \tan\varphi (\tan\alpha -\tan\theta ) \end{array}$$ (10)

According to Eq. (8), the sliding force is a quadratic function of the variable x, and its axis of symmetry is located as follows:


$$x_{0}=-\frac{K_{2}}{2K_{1}}$$ (11)

#### Type II—water-filled rear-edge vertical fissure with unblocked drainage.

As illustrated in Fig 1(b), by resolving all acting forces along the slope inclination and substituting Eqs. (2)–(6) and (12) into Eq. (1), the residual sliding force (Fr_2_) can be expressed as follows:


$$F_{r}=K_{1}x^{2}+K_{2}x+\frac{1.35}{\text{2}}\gamma_{w}H^{2}\cos\theta +\frac{\text{1}}{\text{2}}\gamma_{w}H^{2}\sin\theta \tan\varphi$$ (12)

$${F_{r}}_{2}=K_{3}x^{2}+K_{4}x+\frac{1.35}{\text{2}}\gamma_{w}H^{2}\cos\theta +\frac{\text{1}}{\text{2}}\gamma_{w}H^{2}\sin\theta \tan\varphi$$ (13)

$$\begin{array}{c} K_{3}=\frac{1.35}{\text{2}}\gamma_{w}(\tan\alpha -\tan\theta {)}^{2}\cos\theta +\frac{\text{1}}{\text{2}}\gamma (\tan\alpha -\tan\theta )\cos\theta (1.35\tan\theta -\tan\varphi )\\ \;+\;\frac{\text{1}}{\text{2}}\gamma_{w}\frac{(\tan\alpha -\tan\theta )\tan\varphi }{\cos\theta }\;+\;\frac{\text{1}}{\text{2}}\gamma_{w}(\tan\alpha -\tan\theta {)}^{2}\sin\theta \tan\varphi \end{array}$$ (14)

$$\begin{array}{c} K_{4}=1.35H\gamma \sin\theta \;+\;1.35\gamma_{W}H(\tan\alpha -\tan\theta )\cos\theta -\frac{c}{\cos\theta }-H\gamma \tan\varphi \cos\theta \\ \;+\;\frac{\gamma_{W}H\tan\varphi }{2\cos\theta }+\gamma_{W}H\sin\theta \tan\varphi (\tan\alpha -\tan\theta ) \end{array}$$ (15)

The axis of symmetry corresponding to Eq. (13) is determined as follows:


$$x_{1}=-\frac{K_{4}}{2K_{3}}$$ (16)

#### Type III—considering only water filling in the rear-edge vertical fissure.

As shown in Fig 1(c), by resolving all acting forces along the slope inclination and substituting Eqs. (2)–(4), (6), and (17) into Eq. (1), the residual sliding force (Fr3) can be expressed as follows:


R=cL+(Gcosθ−Vsinθ)tanφ (17)

$$Fr_{\text{3}}=K_{5}x^{2}+K_{6}x+\frac{1.35}{\text{2}}\gamma_{w}H^{2}\cos\theta +\frac{\text{1}}{\text{2}}\gamma_{w}H^{2}\sin\theta \tan\varphi$$ (18)

$$\begin{array}{c} K_{5}=\frac{\text{1}}{\text{2}}\gamma_{w}(\tan\alpha -\tan\theta {)}^{2}\sin\theta \tan\varphi \;+\;\frac{1.35}{\text{2}}\gamma_{w}(\tan\alpha -\tan\theta {)}^{2}\cos\theta \\ \;\;+\;\frac{\text{1}}{\text{2}}\gamma (\tan\alpha -\tan\theta )\cos\theta (1.35\tan\theta -\tan\varphi ) \end{array}$$ (19)

$$\begin{array}{c} K_{6}=1.35H\gamma \sin\theta -H\gamma \tan\varphi \cos\theta +1.35\gamma_{w}H(\tan\alpha -\tan\theta )\cos\theta \\ \;+\;\gamma_{w}H\sin\theta \tan\varphi (\tan\alpha -\tan\theta )-\frac{c}{\cos\theta } \end{array}$$ (20)

The axis of symmetry corresponding to Eq. (18) is determined as follows:


$$x_{2}=-\frac{K_{6}}{2K_{5}}$$ (21)

#### Type IV—neglecting water pressure effects.

As illustrated in Fig 1 (d), by resolving all acting forces along the slope inclination and substituting Eqs. (3)–(4), (22), and (23) into Eq. (1), the residual sliding force (Fr4) can be expressed as follows:


T=Gsinθ (22)

R=cL+Gcosθtanφ (23)

$${F_{r}}_{\text{4}}=K_{7}x^{2}+K_{8}x$$ (24)

$$K_{\text{7}}=\frac{\text{1}}{\text{2}}\gamma (\tan\alpha -\tan\theta )\cos\theta (1.35\tan\theta -\tan\varphi )$$ (25)

$$K_{8}=1.35H\gamma \sin\theta -\frac{c}{\cos\theta }-H\gamma \tan\varphi \cos\theta$$ (26)

The axis of symmetry corresponding to Eq. (24) is determined as follows:


$$x_{3}=-\frac{K_{8}}{2K_{7}}$$ (27)

### Solution of extremum points for sliding force and sensitivity analysis of parameters

As shown in Eqs. (8), (13), (18), and (24), the residual sliding force (Fr) in all four scenarios (I – IV) exhibits a quadratic dependence on the width (x) of the potential slip surface. When k_1_, k_3_, k_5_, and k_7_ are all less than zero, the quadratic curves open downward, and the residual sliding force Fr attains its maximum at x_0_ =−k_2_/2k_1_, x_1_ =−k_4_/2k_3_, x_2_ =−k_6_/2k_5_, and x_3_ = −k_8_/2k_7_, respectively. If the extremum point lies at x < 0, the residual sliding force reaches its maximum at the slope face. For 0 < x < H/ (tanθ−tanα), the residual sliding force attains its peak at x_0_ =−k_2_/2k_1_, x_1_ =−k_4_/2k_3_, x_2_ =−k_6_/2k_5_, and x_3_ = −k_8_/2k_7_. When x > H/ (tanθ−tanα), the residual sliding force reaches its highest value near the rear edge of the slope.

When k_1_, k_3_, k_5_,and k_7_ are positive, each quadratic curve opens upward, and the residual sliding force (F_r_) reaches its minimum at x_0_=−k_2_/2k_1_, x_1_=−k_4_/2k_3_, x_2_=−k_6_/2k_5_, and x_3_=−k_8_/2k_7_, respectively. If the extremum point occurs at x < 0, the residual sliding force reaches its maximum near the rear-edge zone of the slope. If 0 < x < H/ (tanθ−tanα), the residual sliding force reaches its maximum either near the slope face or close to the rear-edge region. When x > H/(tanθ−tanα), the residual sliding force reaches its maximum at the slope face.

As shown in Eqs. (9), (14), (19), and (25), the coefficients k_1_, k_3_, k_5_, and k_7_ become negative only when *α* < *φ* < *θ* or *φ* < *α* < *θ*. Conversely, for *α* < *θ* < *φ*, *θ* < *φ* < *α*, *φ* < *θ* < *α*, or *θ* < *α* < *φ*, these coefficients remain positive.

When k_1_, k_3_, k_5_, and k_7_ are positive, the quadratic curves open upward, and the maximum residual sliding force occurs near either the slope face or the rear-edge region of the slope. As the slope height (*H*), crest inclination (*α*), bedding dip angle (*θ*), and shear strength parameters (*c, φ*) vary, the maximum residual sliding force for all four scenarios (I–IV) consistently occurs at these two locations. Therefore, further discussion is deemed unnecessary and is not elaborated upon in this study.

### Factors influencing the extremum of residual sliding force (*φ* < *α* < *θ*)

**Fig 2:**
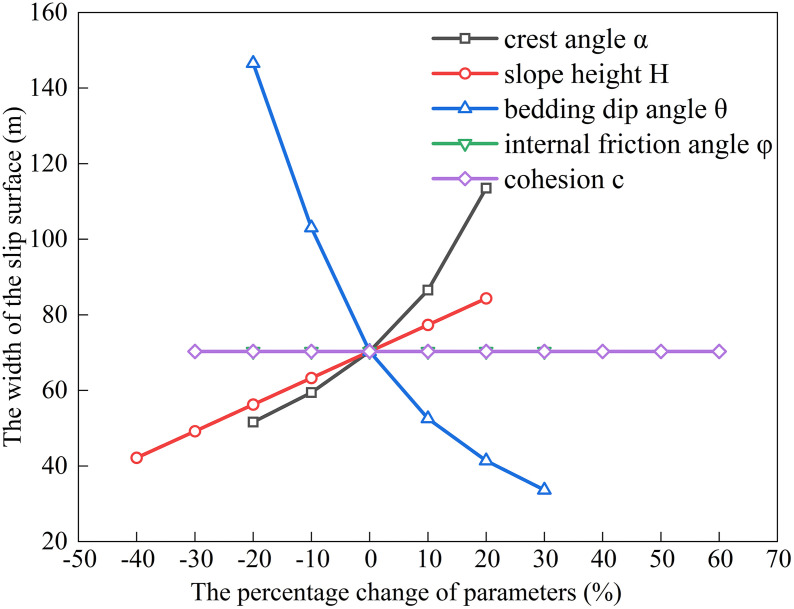
Analysis of Parameter Sensitivity for Type I.

**Fig 3:**
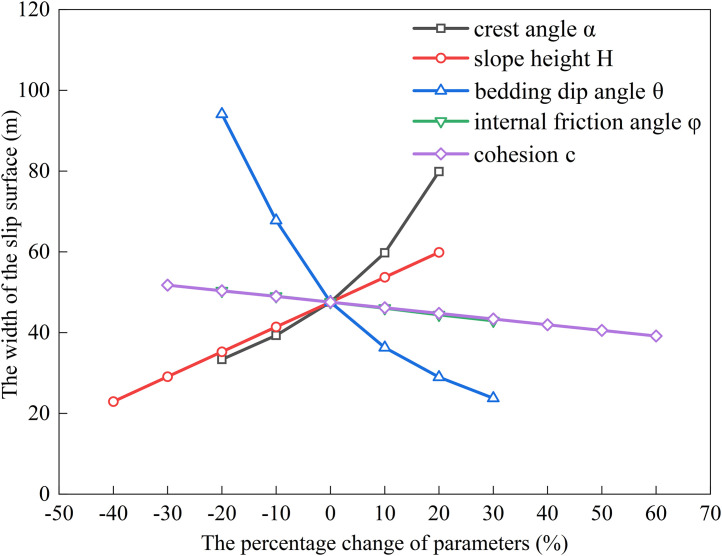
Analysis of Parameter Sensitivity for Type II.

**Fig 4:**
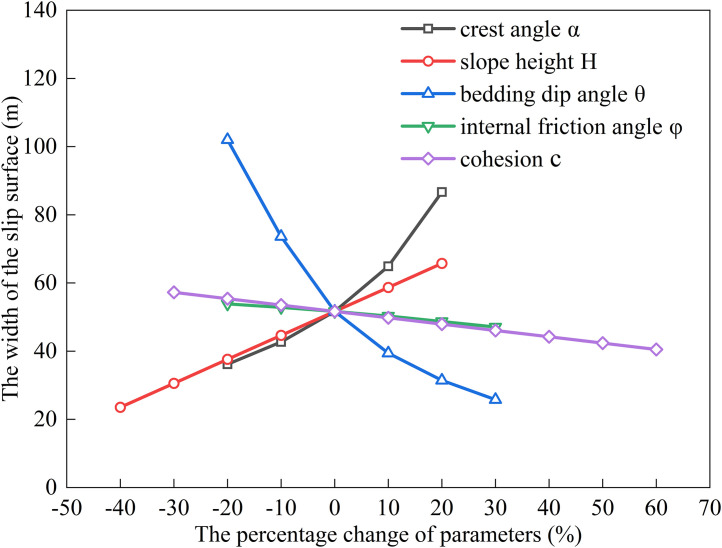
Analysis of Parameter Sensitivity for Type III.

**Fig 5:**
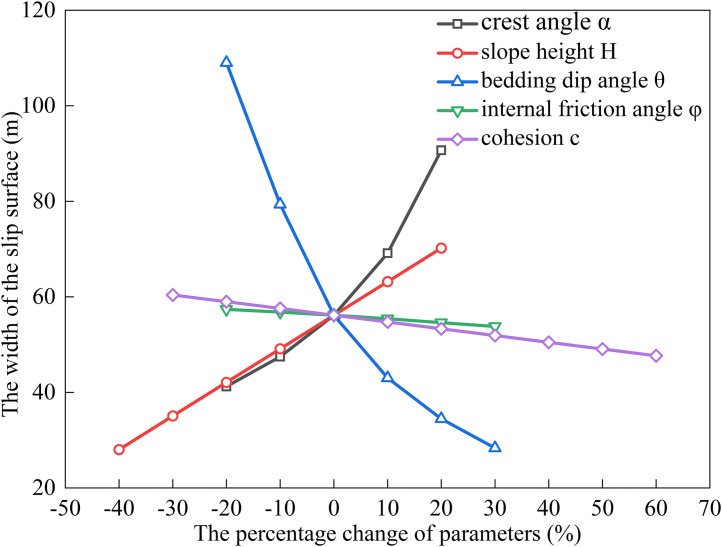
Analysis of Parameter Sensitivity for Type IV.

The bedding dip (*θ*) for gently inclined strata typically ranges from 10^°^ to 40^°^ [30].Using baseline parameters of *H* = 15 m, *α* = 20^°^, *θ* = 30^°^, *c* = 30 kPa, *φ* = 14^°^, rock unit weight (*γ*) = 25 kN/m³, and water unit weight (*γ*_w_) = 10 kN/m³, we substitute these into Eqs. (11), (16), (21), and (27) to analyze the dependence of the potential sliding surface width on *H*, *φ*, *θ*, *c*, and *α* (see Figs 2, 3, 4, 5). The selected parameters comply with the requirements of most engineering designs and align with the empirical values employed in relevant engineering cases, thereby ensuring the practical relevance and applicability of the model.

### Factors influencing the extremum of residual sliding force (*α* < *φ* < *θ*)

**Fig 6:**
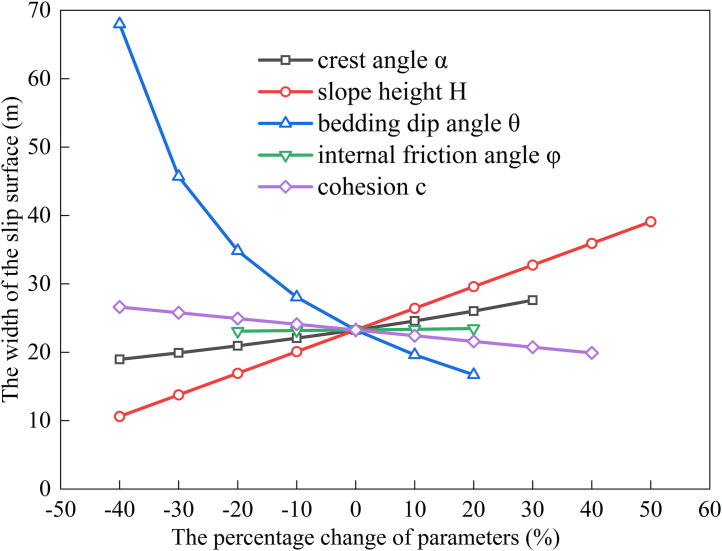
Analysis of Parameter Sensitivity for Type I.

**Fig 7:**
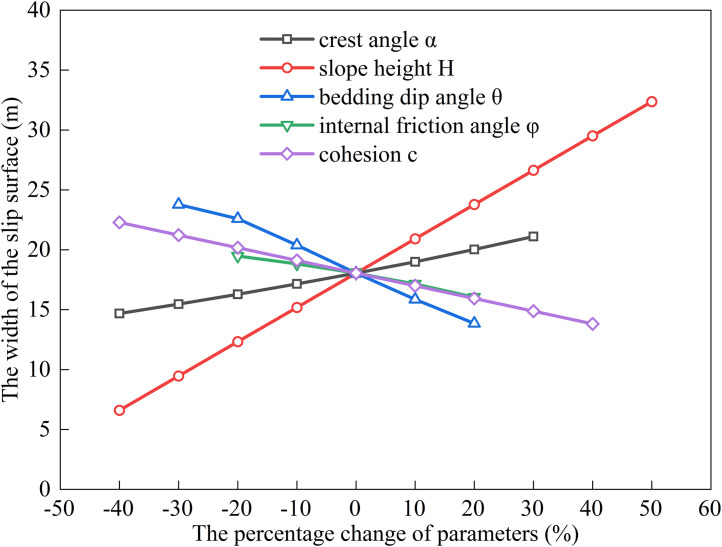
Analysis of Parameter Sensitivity for Type II.

**Fig 8:**
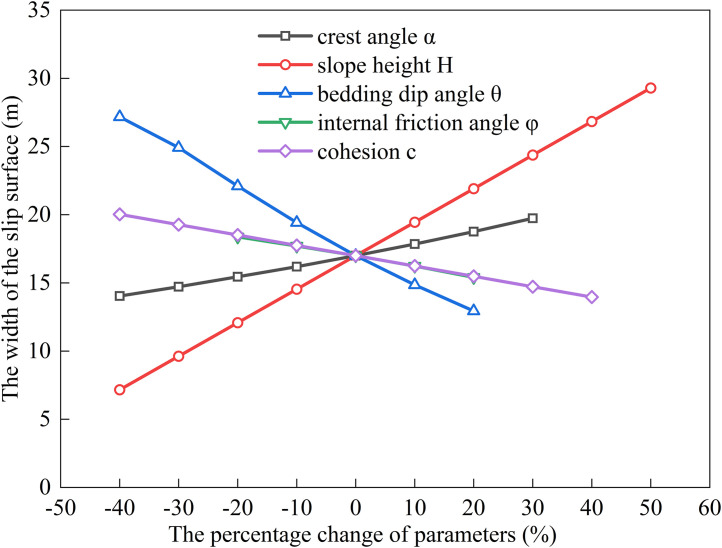
Analysis of Parameter Sensitivity for Type III.

**Fig 9:**
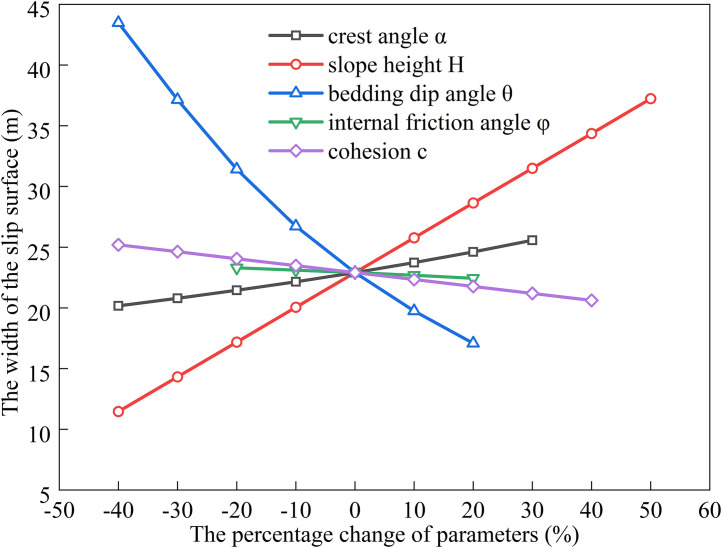
Analysis of Parameter Sensitivity for Type IV.

The relationship between the sliding surface width and slope height (*H*), bedding dip angle (*θ*), internal friction angle (*φ*), cohesion (*c*), and slope crest inclination (*α*) was analyzed by substituting the following baseline parameters into Eqs. (11), (16), (20), and (24): slope height *H* = 15 m, crest inclination *α* = 10^°^, shear strength parameters *c* = 35 kPa, *φ* = 14^°^, bedding dip angle *θ* = 35^°^, rock unit weight *γ* = 25 kN/m³, and water unit weight *γ*_w_ = 10 kN/m³. The results are shown in Figs 6, 7, 8, 9.

Based on Figs 2, 3, 4, 5, 6, 7, 8, 9, the variation in the width of the slip surface is mainly controlled by the combined effects of geometric factors and shear strength parameters. The increase in slope height (*H*) and crest inclination (*α*) significantly amplifies the driving force component of the slope’s self-weight. However, the corresponding increase in frictional resistance along the structural plane is insufficient to counteract this effect, thereby leading to an expansion of the sliding surface. The increase in bedding dip angle (*θ*) elevates the normal stress along the sliding surface, thereby enhancing frictional resistance and constraining the expansion of the slip surface, which leads to a gradual reduction in width. The influence of shear strength parameters is relatively weak but shows variability depending on the parameter combinations. When the condition *φ* < *α* < *θ* is met, the sliding mass experiences insufficient frictional resistance, and the instability mechanism is primarily controlled by fissure water pressure and geometric effects. The influence of *φ* and *c* is not significant, and the width only decreases as the parameters increase under Type II–IV conditions. Under the condition *α* < *φ* < *θ*, the frictional effect becomes dominant, and increases in *φ* and *c* significantly improve the resisting capacity, resulting in a narrower slip surface. However, in Type I conditions, the blockage of drainage paths leads to concentrated water pressure, significantly weakening the effective stress, and resulting in an anomalous trend where the width increases as *φ* increases. In general, the sliding surface width is highly sensitive to geometric parameters (*H*, *α*, *θ*), while its sensitivity to shear strength parameters (*c*, *φ*) is relatively lower. Furthermore, the pore-water pressure boundary conditions determine the positive or negative effect of *φ* on the variation trend of the slip surface width. This reflects the combined influence of geometric, hydraulic, and strength factors in controlling the instability mechanism of gently inclined bedding rock slopes. Additionally, the boundary condition of pore-water pressure dictates the positive and negative effects of *φ* on the width variation, highlighting the combined influence of geometry, hydraulics, and strength in controlling the failure mechanism of gently inclined layered rock slopes.

### Case study analysis

To evaluate the applicability of the proposed formulations, parametric trial calculations were conducted using representative engineering values [30]. Baseline parameters include *α* = 10^°^, *γ*_w_ = 10 kN/m^3^, *γ* = 25 kN/m^3^, *θ* = 30^°^, with slope height (*H*) varying from 6 to 30 m. According to the Technical Code for Building Slope Engineering [31], the admissible ranges for cohesive strength (*c*) are 20–50 kPa and for internal friction angle (*φ*) are 12^°^–18^°^.

The following performance indices are defined: η_1_ = (Fr_1_ − Fr_4_)/Fr_1_, η_2_ = (Fr_2_ − Fr_4_)/Fr_2_ and η_3_= (Fr_3_ − Fr_4_)/Fr_3_. Three representative parameter sets are then examined: {*c* = 30 kPa, *φ* = 12^°^}, {*c* = 40 kPa, *φ* = 15^°^}, and {*c* = 50 kPa, *φ* = 18^°^}. The safety factor and residual sliding force at the most adverse rear-edge vertical fissure location for Types I–IV (Conditions I–IV) are summarized in Table 1. The safety factor is defined as the ratio of the resisting force to the driving force along a sliding surface, and the residual sliding force constitutes part of the driving force. An increase in the residual sliding force elevates the total driving force, thereby influencing the calculated value of the safety factor. Specifically, a rise in the residual sliding force leads to a decrease in the safety factor, which consequently heightens the risk of slope instability. Therefore, the calculation of the residual sliding force can serve as a supplementary evaluation tool for assessing the safety factor, contributing to the overall judgment of slope stability.

**Table 1:** Comparison table of residual sliding force and slope safety factor.

strength parameters	Height of the slope H/m	Residual sliding force Fr/(kN/m)				Safety stability factor Fs				Relative deviation		
		I	II	III	IV	I	II	III	IV	η_1_	η_2_	η_3_
c = 30kPa φ = 12°	6	307.1	261.4	241.6	154.5	0.65	0.60	0.54	0.99	49.7	40.9	36.1
	9	909.7	732.8	671.1	584	0.57	0.62	0.68	0.84	35.8	20.3	13.0
	12	1870.1	1483	1375.9	1288.8	0.49	0.59	0.66	0.74	31.1	13.1	6.3
	15	3188.3	2512.3	2356.3	2269.2	0.43	0.56	0.62	0.68	28.8	9.7	3.7
	18	4864.3	3820.4	3612	3524.9	0.38	0.53	0.60	0.63	27.5	7.7	2.4
	21	6898.1	5407.6	5143.2	5056.1	0.34	0.51	0.57	0.60	26.7	6.5	1.7
	24	9289.7	7273.7	6949.9	6862.8	0.31	0.49	0.55	0.58	26.1	5.7	1.3
	27	12039	9418.7	9032	8944.9	0.29	0.48	0.54	0.55	25.7	5.03	0.9
	30	15146	11842	11389	11302	0.27	0.47	0.52	0.54	25.4	4.6	0.8
c = 40kPa φ = 15°	6	243.7	235	260.8	46.2	0.47	0.07	0.10	1.19	81.1	80.4	82.3
	9	721.0	585.6	536.6	321.9	0.65	0.57	0.39	1.03	55.3	45.0	40.0
	12	1552.5	1190.7	1060.9	846.3	0.60	0.63	0.66	0.92	45.5	28.9	20.2
	15	2737.8	2050.3	1833.7	1619.1	0.54	0.63	0.70	0.85	40.8	21.0	11.7
	18	4277.1	3164.5	2855.1	2640.4	0.49	0.61	0.69	0.80	38.3	16.6	7.5
	21	6170.4	4533.2	4125	3910.3	0.44	0.60	0.68	0.76	36.6	13.7	5.2
	24	8417.6	6156.4	5643.4	5428.7	0.41	0.58	0.67	0.73	35.5	11.8	3.8
	27	11018	8034.1	7410.3	7195.6	0.38	0.57	0.66	0.70	34.7	10.4	2.9
	30	13974	10166	9425.7	9211.1	0.35	0.56	0.64	0.68	34.1	9.4	2.3
c = 50kPa φ = 18°	6	239.7	239.7	239.7	0.22	0.06	0.09	0.12	1.34	99.9	99.9	99.9
	9	590.9	539.3	539.3	120.4	0.60	0.03	0.06	1.18	79.6	77.7	77.7
	12	1280.7	1012	958.8	461.5	0.67	0.49	0.01	1.08	64.0	54.4	51.8
	15	2321.8	1715.2	1524.5	1023.3	0.63	0.60	0.43	1.01	55.9	40.3	32.9
	18	3714.2	2648.7	2307.2	1806.0	0.58	0.63	0.63	0.95	51.4	31.8	21.7
	21	5457.9	3812.7	3310.8	2809.6	0.54	0.63	0.69	0.91	48.5	26.3	15.1
	24	7552.9	5207	4535.2	4034.0	0.50	0.63	0.71	0.87	46.6	22.5	11.1
	27	9999.4	6831.8	5980.4	5479.2	0.47	0.62	0.72	0.84	45.2	19.8	8.4
	30	12797	8686.9	7646.5	7145.3	0.44	0.62	0.73	0.82	44.2	17.8	6.6

The residual sliding force for Type IV was compared with those of Types I–III. As shown in Table 1, the relative deviations in residual sliding force are η_1_ = 25.4% − 99.9% (Type I vs. IV), η_2_ = 4.6% − 99.9% (Type II vs. IV) and η_3_ = 0.8% − 99.9% (Type III vs. IV).A significant difference in slope stability coefficient is observed between models that include and those that neglect rear -edge vertical fissure water pressure. Therefore, neglecting rear -edge vertical fissure water pressure may result in an underestimation of slope failure risk, potentially compromising the overall safety of slope engineering.

### Engineering case study and verification analysis

### Representative engineering case study

**Fig 10:**
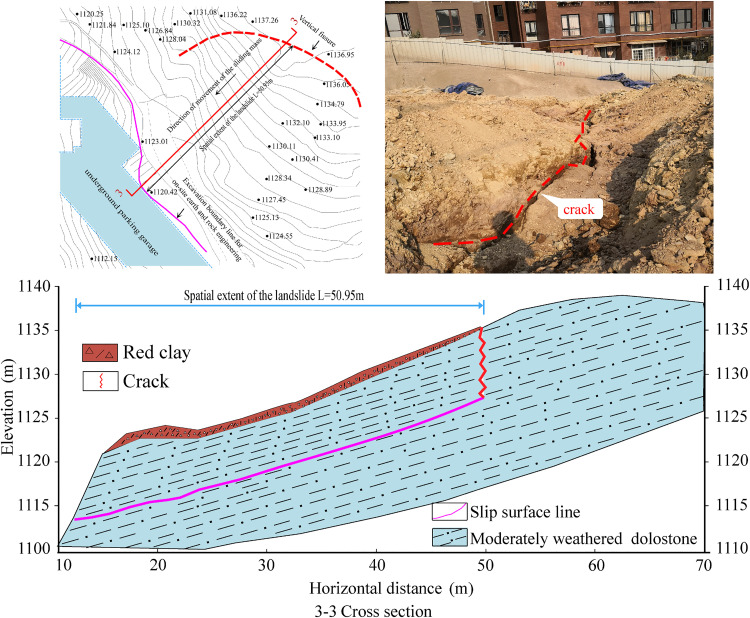
Plan view and cross-section 3−3 of the landslide area in Wudang District, Guiyang City.

In the course of the slope support engineering for the Beiya Shangcheng B District construction project in Wudang District, Guiyang, adverse geological conditions, such as interlayer mudstone, led to downslope sliding along the interlayer mudstone when the excavation reached the fire lane terrace elevation of 1110 m, exposing the surface. Surface deformation monitoring indicated that the landslide extended approximately 70m in the longitudinal direction and 60 m along the primary sliding path. The shear outlet was located approximately 6.7m below the slope crest, where pronounced seepage occurred due to intense rainfall infiltration. Tensile cracks were observed near the slope crest, approximately 51–52 m horizontally from the slope face, with a width of about 0.1m (see Fig 10).

The slope-forming rock mass primarily consists of thin-bedded purplish-red dolomite and medium-thick grayish-brown argillaceous dolomite from the Lower Triassic Anshun Formation, with a bedding attitude of 210^°^∠16^°^.The upper part of the slope is locally covered with plain fill and plastic red clay. According to the in-situ geological survey report, the shear strength parameters of the structural plane are as follows: cohesion (*c*) = 21.95 kPa, internal friction angle (*φ*) = 6.35^°^, saturated unit weight of the rock mass (γ) = 24.1 kN/m³, and water unit weight (γ_w_) = 10 kN/m³.Considering the slope geometry (*θ* = 16^°^, *α* = 13.1^°^) and the fact that the drainage outlet at the slope toe remained unobstructed, the Type I failure mode (blocked drainage condition) was excluded from the analysis. Using the developed Type II–IV failure models.Eqs. (16), (21), and (27) were applied to identify the critical locations of rear-edge vertical fissures corresponding to the extremum of residual sliding force under different hydraulic conditions.


$$x_{1}=-\frac{K_{4}}{2K_{3}}=51.61m$$ (28)

$$x_{2}=-\frac{K_{6}}{2K_{5}}=49.49m$$ (29)

$$x_{3}=-\frac{K_{8}}{2K_{7}}=57\mathrm{.86}m$$ (30)

The horizontal distances from the rear-edge vertical fissure to the shear outlet, corresponding to the maximum residual sliding force, were calculated as 51.61 m (Type II), 49.49 m (Type III), and 57.86 m (Type IV). The results from Type II (51.61 m) and Type III (49.49 m) models show excellent agreement with the field-measured distance of 50–51 m, thereby validating the reliability of these models. In contrast, the significant deviation in the Type IV result underscores the invalid assumption of neglecting water pressure under the actual, saturated conditions.

Using the field-measured horizontal distance between the rear-edge vertical fissure and the shear outlet (~50.95 m) and the measured slope parameters as baseline inputs, the residual sliding force, active rock pressure, and safety factor of the sliding block were computed for Types II–IV, as summarized in Table 2. Active rock pressure is primarily related to the lateral pressure on structural planes and is typically used for calculating the stability of retaining structures. In contrast, the residual sliding force reflects the difference between the driving and resisting forces along a sliding surface and is mainly employed to assess the risk of slope instability. Although these two concepts differ in their physical implications and application scenarios, they play complementary roles in slope stability analysis and support design. A comprehensive analysis of both active rock pressure and residual sliding force can provide a more thorough and reliable basis for slope stability evaluation and reinforcement design.

**Table 2:** Calculation of Factor of Safety, residual sliding force and active rock pressure.

Type of model	Residual sliding force/(kN/m)	Active rock pressure/(kN/m)	Safety stability factor
Type II	132.71	133.79	0.93
Type III	16.32	16.45	0.99
Type IV	−60.94	−61.44	1.03

As shown by the stability analysis in Table 2, (1) under non-water-filled conditions, both active rock pressure and residual sliding force are negative, indicating that the slope remains naturally stable. (2) When rear-fissure water filling is considered, the active rock pressures for Type II and Type III modes increase to 133.8 kN/m and 16.5 kN/m, respectively, with corresponding residual sliding forces of 132.7 kN/m and 16.3 kN/m, indicating the onset of instability consistent with the observed landslide behavior. Following the landslide, emergency mitigation measures, including toe counter-pressure filling, surface plastic-sheet waterproofing, and real-time monitoring, were promptly implemented by the responsible engineering units.

### Case study and analytical evaluation

Analysis of the Beiya Shangcheng Zone B slope project in Wudang District, Guiyang, suggests that stability evaluation must explicitly incorporate the effects of rear-edge vertical fissures under water-filled conditions. For very gently inclined slopes, neglecting fissure water pressure can lead to an overestimation of the stability factor by approximately 18%–22%, as shown by back-calculated results from the case study. The hydraulic gradient can reduce the effective stress along weak structural interfaces by 30%–45%, significantly increasing the likelihood of slope sliding. Moreover, identifying the most critical location of rear-edge vertical fissures is crucial for defining accurate computational boundaries and preventing engineering failures due to the omission or misestimation of residual sliding force.

The primary triggering factor of the landslide was insufficient and delayed slope support during earthwork excavation, leading to toe cutting of the dip slope and subsequent sliding along the interbedded softened clay layer. The softened clay interlayer extensively developed between strata with varying thickness, acts as the principal weak structural surface that critically governs slope stability. The slope, exhibiting a dip-slope configuration, fails primarily through translational sliding along the weak structural interface.

The instability of gently inclined bedding slopes is closely linked to the evolution of steep unloading fissures at the slope crest, whose formation and propagation constitute a progressive process of structural degradation. During the geotechnical investigation phase, if slope excavation is incomplete or recently initiated, unloading fissures may not have fully developed, which could lead to an inaccurate assessment of the slope’s stability, not reflecting its behavior in service or under design conditions. When rear-edge vertical fissures are opened by unloading effects, immediate sealing is recommended to prevent water ingress, and drainage holes should be installed within the slope to alleviate pore-water pressure and improve stability.

## Conclusion

This study tackles the difficulty in stability assessment of gently dipping bedding rock slopes caused by uncertain rear fissure locations. It presents a sliding-force calculation method and a criterion for determining the most unfavorable fissure position, backed by theoretical derivation and case studies. The primary conclusions are:

(1) Engineering case validation confirms the rationality of the proposed sliding-force model. Our analysis indicates that accurate stability assessment necessitates explicit consideration of the rear fissure location and its water pressure conditions. Omitting these influences introduces significant deviations in the residual sliding force and can critically underestimate the potential for slope instability.
(2) Sensitivity analysis shows that the sliding-surface width is highly responsive to variations in geometric parameters—specifically crest inclination (*α*), slope height (*H*), and bedding dip angle (*θ*)—but exhibits comparatively low sensitivity to rock mass shear strength parameters (*c*, *φ*). This finding provides a clear basis for identifying and prioritizing key parameters in slope hazard investigation and risk assessment.
(3) This study elucidates the occurrence pattern of the most critical sliding surface under different geometric parameter combinations. When the slope crest inclination (*α*), bedding dip angle (*θ*), and internal friction angle (*φ*) satisfy specific relationships (e.g.,*α* < *θ* < *φ*), the maximum residual sliding force tends to occur at either the slope face or the rear-edge region. Its theoretical extremum can be approximated by 0.675γ_w_H^2^cosθ+0.5γ_w_H^2^sinθtanφ. This relationship provides a practical basis for quickly identifying the most probable failure mode in gently dipping layered rock slopes.
(4) In the design and construction of gently inclined layered rock slopes, the primary focus should be on rigorously controlling unloading-induced deformation and ensuring that stabilization structures (e.g., anti-slide piles and anchors) have sufficient anchorage depth. Furthermore, an efficient drainage system must be incorporated to dissipate fissure water pressure. This measure is fundamental for preventing landslides triggered by high water pressure at the source.

This study has certain limitations regarding its generalizability. Specifically, the model is based on static equilibrium theory and assumes a vertically oriented and fully saturated trailing fissure. Consequently, the findings may not be applicable to more complex engineering scenarios involving non-vertical fissures, partially saturated conditions, or transient seepage processes. Additionally, the model validation relied on only a single engineering case. While this provides preliminary verification of the model’s effectiveness, it is insufficient to fully validate its applicability under diverse geological and hydrological conditions. Future work should aim to extend the model to accommodate non-vertical or stepped fissures, unsaturated conditions, and transient seepage. Furthermore, systematic validation using multiple slope cases from different regions and geological settings should be conducted. The integration of probabilistic analysis and numerical simulation techniques is also recommended to quantify parameter uncertainties and enhance the reliability and practical applicability of the proposed method.

## References

[1] Liu J, Xu Q, Wang S, Siva Subramanian S, Wang L, Qi X. Formation and chemo-mechanical characteristics of weak clay interlayers between alternative mudstone and sandstone sequence of gently inclined landslides in Nanjiang, SW China. Bull Eng Geol Environ. 2020;79(9):4701–15. doi: 10.1007/s10064-020-01859-y

[2] Yi S, Chen J, Pan J, Huang J, Qiu Y. Risk assessment of a layered slope considering spatial variabilities of interlayer and intralayer. Computers and Geotechnics. 2023;156:105236. doi: 10.1016/j.compgeo.2022.105236

[3] Sun SW, Li Y, Yang XR. Study on the failure mechanism of rock slopes with dipped layered structures under various rock dip conditions. Chinese Journal of Rock Mechanics and Engineering. 2024;43(7):1607–20. doi: 10.13722/j.cnki.jrme.2023.1109

[4] Xu Q, Wang W, Li L, Cao Y. Failure mechanism of gently inclined shallow landslides along the soil-bedrock interface on ring shear tests. Bull Eng Geol Environ. 2021;80(5):3733–46. doi: 10.1007/s10064-021-02171-z

[5] Nie L, Li Z, Zhang M, Xu L. Deformation characteristics and mechanism of the landslide in West Open-Pit Mine, Fushun, China. Arab J Geosci. 2014;8(7):4457–68. doi: 10.1007/s12517-014-1560-2

[6] Lin F, Wu LZ, Huang RQ, Zhang H. Formation and characteristics of the Xiaoba landslide in Fuquan, Guizhou, China. Landslides. 2017;15(4):669–81. doi: 10.1007/s10346-017-0897-5

[7] Sun H-Y, Pan P, Lü Q, Wei Z-L, Xie W, Zhan W. A case study of a rainfall-induced landslide involving weak interlayer and its treatment using the siphon drainage method. Bull Eng Geol Environ. 2018;78(6):4063–74. doi: 10.1007/s10064-018-1365-8

[8] Tang H, Zou Z, Xiong C, Wu Y, Hu X, Wang L, et al. An evolution model of large consequent bedding rockslides, with particular reference to the Jiweishan rockslide in Southwest China. Engineering Geology. 2015;186:17–27. doi: 10.1016/j.enggeo.2014.08.021

[9] Dai Z, Zhang L, Wang Y, Jiang Z, Xu S. Deformation and failure response characteristics and stability analysis of bedding rock slope after underground adverse slope mining. Bull Eng Geol Environ. 2021;80(6):4405–22. doi: 10.1007/s10064-021-02258-7

[10] Li J, Hu B, Sheng J, Zhang Z. Failure mechanism and treatment of mine landslide with gently-inclined weak interlayer: a case study of Laoyingzui landslide in Emei, Sichuan, China. Geomech Geophys Geo-energ Geo-resour. 2024;10(1). doi: 10.1007/s40948-024-00775-9

[11] Zhong J, Mao Z, Ni W, Zhang J, Liu G, Zhang J, et al. Analysis of Formation Mechanism of Slightly Inclined Bedding Mudstone Landslide in Coal Mining Subsidence Area Based on Finite–Discrete Element Method. Mathematics. 2022;10(21):3995. doi: 10.3390/math10213995

[12] Li XZ, Xu Q. Application of the SSPC method in the stability assessment of highway rock slopes in the Yunnan province of China. Bull Eng Geol Environ. 2015;75(2):551–62. doi: 10.1007/s10064-015-0792-z

[13] Chimidi G, Raghuvanshi TK, Suryabhagavan KV. Landslide hazard evaluation and zonation in and around Gimbi town, western Ethiopia—a GIS-based statistical approach. Appl Geomat. 2017;9(4):219–36. doi: 10.1007/s12518-017-0195-x

[14] Tao T, Shi W, Liang F, Wang X. Failure mechanism and evolution of the Jinhaihu landslide in Bijie City, China, on January 3, 2022. Landslides. 2022;19(11):2727–36. doi: 10.1007/s10346-022-01957-w

[15] Chi EA, Tao TJ, Zhao MS, Kang Q. Failure Mode Analysis of Bedding Rock Slope Affected by Rock Mass Structural Plane. AMM. 2014;602–605:594–7. doi: 10.4028/www.scientific.net/amm.602-605.594

[16] Mallick J, Singh RK, AlAwadh MA, Islam S, Khan RA, Qureshi MN. GIS-based landslide susceptibility evaluation using fuzzy-AHP multi-criteria decision-making techniques in the Abha Watershed, Saudi Arabia. Environ Earth Sci. 2018;77(7). doi: 10.1007/s12665-018-7451-1

[17] Liu W, Zhang Y, Liang Y, Sun P, Li Y, Su X, et al. Landslide Risk Assessment Using a Combined Approach Based on InSAR and Random Forest. Remote Sensing. 2022;14(9):2131. doi: 10.3390/rs14092131

[18] Chen Z, Zhou H, Ye F, Liu B, Fu W. The characteristics, induced factors, and formation mechanism of the 2018 Baige landslide in Jinsha River, Southwest China. CATENA. 2021;203:105337. doi: 10.1016/j.catena.2021.105337

[19] Yuan H. Exploration of maximum residual sliding force calculation for bedding rock slopes. Low Temperature Building Technology. 2016;38(1):125–7. doi: 10.13905/j.cnki.dwjz.2016.01.046

[20] Cui S, Pei X, Huang R. Effects of geological and tectonic characteristics on the earthquake-triggered Daguangbao landslide, China. Landslides. 2017;15(4):649–67. doi: 10.1007/s10346-017-0899-3

[21] Yang Y, Xing H, Yang X, Chen M. Experimental study on the dynamic response and stability of bedding rock slopes with weak interlayers under heavy rainfall. Environ Earth Sci. 2023. doi: 10.1007/s12665-018-7624-y

[22] Yu H, Li C, Zhou J-Q, Chen W, Long J, Wang X, et al. Recent rainfall- and excavation-induced bedding rockslide occurring on 22 October 2018 along the Jian-En expressway, Hubei, China. Landslides. 2020;17(11):2619–29. doi: 10.1007/s10346-020-01468-6

[23] Li Y, Wang SP, Zhang ZH, Li SJ. Study on the Relationship between Factor of Safety and Angle of Dip of Bedding Rock Slope when Exposed to Reservoir Water Level Fluctuation. Advanced Materials Research. 2023. doi: 10.4028/www.scientific.net/amr.446-449.3894

[24] Hoek E, Bray JD. Rock slope engineering. London: Revised Second Edition. 1977.

[25] Xia KZ, Chen CX, Liu XM. Analysis of sliding failure mechanism of gently inclined bedding compound rock mass slope under hydraulic pressure. Chinese Journal of Rock Mechanics and Engineering. 25AD. doi: 10.13722/j.cnki.jrme.2014.s2.046

[26] Tan LJ, Zhang HN, Sheng HW. Mechanism of Bedding-Slip Failure of Gently Inclined Rock Slope Due to Hydraulic Pressure. Journal of Yangtze River Scientific Research Institute. 2014;31(9):47–53. 10.3969/j.issn.1001-5485.2014.09.009

[27] Zhao L, Cao J, Zhang Y, Luo Q. Effect of hydraulic distribution on the stability of a plane slide rock slope under the nonlinear Barton-Bandis failure criterion. Geomech Eng. 2015;8(3):391–414.

[28] Li WB, Li KG, Wu S. Effect of Slope Surface Tension Crack Filling on the Stability of Plane Sliding Slope. Nonferrous Metals Engineering. 2022;12(7):163–71.

[29] Wu HB, He ZP, Cao WW. Study on the stability of plane sliding rock slopes based on different water pressure distributions. Geotechnical Mechanics. 2011;32(8):2493–9. 10.3969/j.issn.1000-7598.2011.08.040

[30] Chen B, Yang W, Xie Q. Calculation of active rock pressure along gently inclined weak outward-dipping structural surfaces. Geotechnical Mechanics. 2023;44(11):3272–9, 3287. 10.16285/j.rsm.2023.0469

[31] Ministry of Housing and Urban-Rural Development of the People’s Republic of China. GB 50330-2013 Technical Code for Building Slope Engineering[S]. Beijing: China Architecture & Building Press, 2014.

